# Supplementary material for: MHConstructor: A high-throughput, haplotype-informed solution to the MHC assembly challenge
Source: bioRxiv. 2024 May 21:2024.05.20.595060. Preprint. [Version 1] doi: 10.1101/2024.05.20.595060 (PMC11142050; doi:10.1101/2024.05.20.595060)
Supplement: Supplement 1 [file media-1.pdf]

Supplemental data

Table of contents:

Results

1. Haplotype-guided, *de novo* short read MHC assembly workflow

2. Evaluation of assembly performance metrics

I. **Supplemental Table 1.** Best kmer size predictions for mappable reads, at given read counts.

II. **Supplemental Table 2.** Best kmer size predictions for un-mappable reads, at given read counts.

III. **Supplemental Figure S1.** Impact of kmer size choice on *de novo* assembly contig metrics, for mapped read k= 41, k=51 and k=61.

IV. **Supplemental Table S3.** User hours to generate assemblies. mappable read k = 49, unmappable read k=21.

V. **Supplemental Table S4.** User time (in minutes) to generate contigs and scaffold assemblies in target-capture short reads across multiple coverage depths.

VI. **Supplemental Figure S2.** Impact of starting read count on *de novo* assembly contig metrics.

3. Quantification of MHConstructor error rates using high-quality MHC reference sequences

VII. **Supplemental Table S5.** Assembly statistics from Norman *et al.*, 2017 MHC reference haplotype Illumina short read dataset. *SRA:* SRP348947, *BioProject:* PRJNA764575.

VIII. **Supplemental table S6.** Reference MHC coverage with contigs generated from reads that mapped to reference genome.

IX. **Supplemental Table S7.** Reference MHC coverage with contigs generated from reads that did not map to initial reference genome.

X. **Supplemental Figure S3.** Dotplot of *Assemblytics* assembly alignments between 1KGP WGS MHConstructor assemblies (y-axes) and their corresponding, phased reference MHC haplotypes (x-axes).

XI. **Supplemental Figure S4.** *Assemblytics* analysis of false SV composition for the MHConstructor *de novo* assembly of HG00621, as compared to phased, reference MHC haplotypes for HG00621.

XII. **Supplemental Figure S5.** *Assemblytics* analysis of false SV composition for the MHConstructor *de novo* assembly of NA19240, as compared to phased, reference MHC haplotypes for NA19240.

XIII. **Supplemental Figure S6.** *Assemblytics* analysis of false SV composition for the MHConstructor *de novo* assembly of HG00621, as compared to phased, reference MHC haplotypes for NA20129.

XIV. **Supplemental Table S8**. Number of error sites in base5-validated *de novo* assemblies that are caused by inclusion of a heterozygous site belonging to the alternative haplotype and those caused by assembly error.

XV. **Supplemental figure S7.** Mauve alignment plots confirm that the two DR haplotypes for heterozygous Individual Z are correctly assembled.

XVI. **Supplemental Figure S8.** HLA Class II structure can be correctly assembled even when an incorrectly-match haplotype is used as the assembly guide, so long as the correct haplotype is used for scaffolding orientation

4. MHConstructor assembly of target-capture 60x and 1000genomes 30x WGS short read MHC data.

XVII. **Supplemental Figure S9**. MHConstructor assembly metrics for target capture and WGS assemblies.

XVIII. **Supplemental Figure S10.** Nucleotide diversity within sequencing reads.

Supplemental Table 1. Best kmer size predictions for mappable reads, at given read counts.

ID	200k	500k	1M	2M	3M
EPIC0046	17	No best k	31	45	51
EPIC0053	19	No best k	25	61	33
EPIC0176	17	No best k	21	35	71
EPIC0242	19	No best k	51	45	71
EPIC0246	27	21	21	57	25
Average	19.8	NA	29.8	48.6	50.2
StDev	4.15	NA	12.54	10.43	21.19

Supplemental Table 2. Best kmer size predictions for un-mappable reads, at given read counts.

ID	200k	500k	1M	2M	3M
EPIC0046	17	33	17	31	31
EPIC0053	31	23	23	31	17
EPIC0176	17	17	23	19	19
EPIC0242	27	19	23	21	23
EPIC0246	36	23	27	21	17
Average	25.6	23	22.6	24.6	21.4
StDev	8.47	6.16	3.58	5.90	5.90

A.

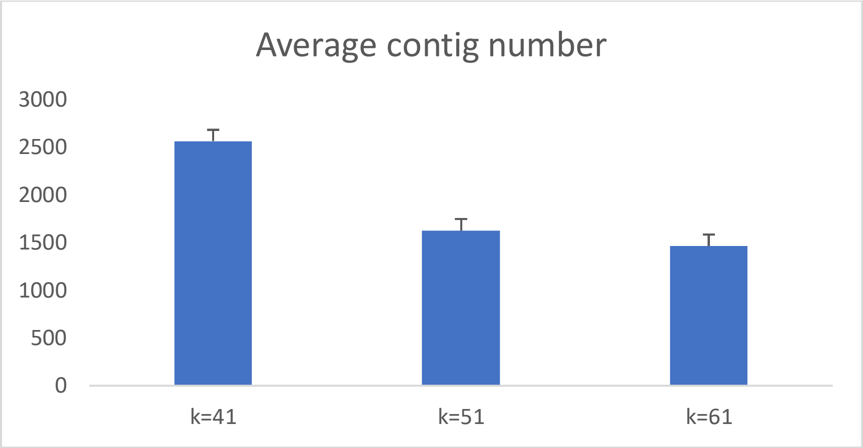

B.

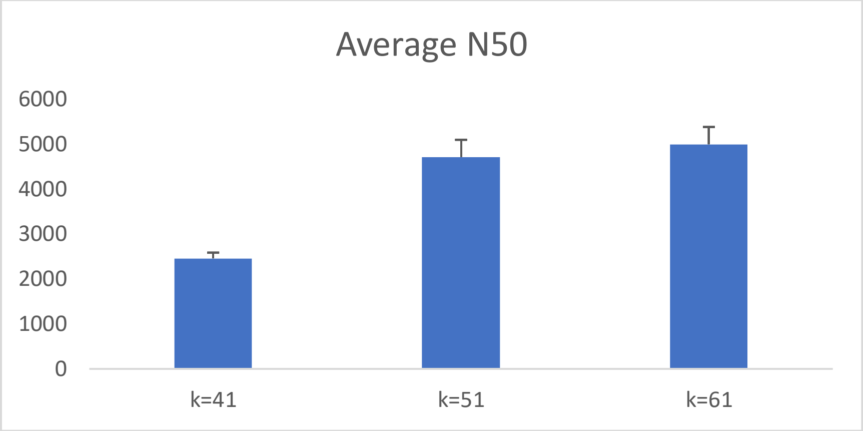

C.

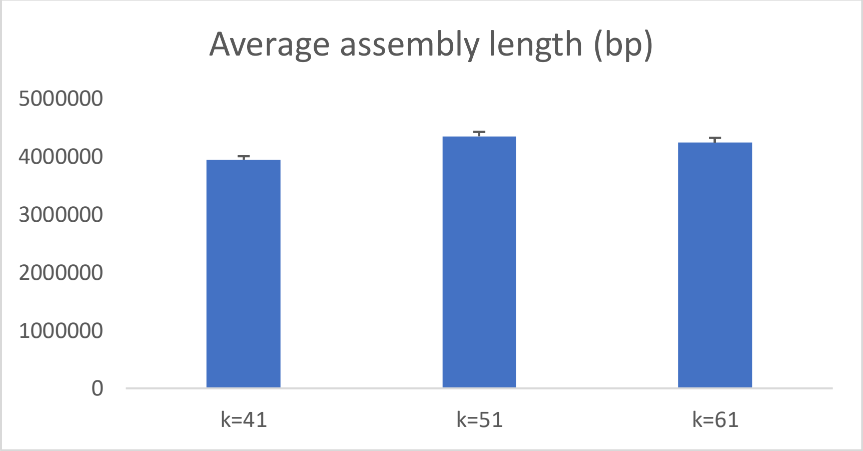

Supplemental Figure S1. Impact of kmer size choice on *de novo* assembly contig metrics, for mapped read k= 41, k=51 and k=61. Test samples subsampled to contain 2 million reads per individual, n=14. A) Average number of contigs generated for each set of subsampled reads. B) Average N50 length (in bp) for each set of subsampled reads. C) Average length (bp) of contigs generated by *de novo* assembly.

Supplemental Table S3. User hours to generate assemblies. mappable read k = 49, unmappable read k=21.

Sample ID	200k	500k	1M	2M	3M
EPIC0046	0.07	0.34	0.89	2.36	6.21
EPIC0053	0.09	0.21	0.66	1.68	3.99
EPIC0176	0.07	0.21	0.65	1.84	6.07
EPIC0242	0.08	0.24	0.55	2.16	2.85
EPIC0246	0.09	0.23	0.57	1.63	4.85
Average	0.08	0.25	0.66	1.93	4.79

Supplemental Table S4. User time (in minutes) to generate contigs and scaffold assemblies in target-capture short reads across multiple coverage depths.

Average coverage	Starting reads	Assembly	Scaffolding+EC	Total
~25X	2 million	36	24	61
~45X	3 million	41	40	81
~60X	4 million	53	51	104

Performed on HPC cluster, 8 threads per sample

A.

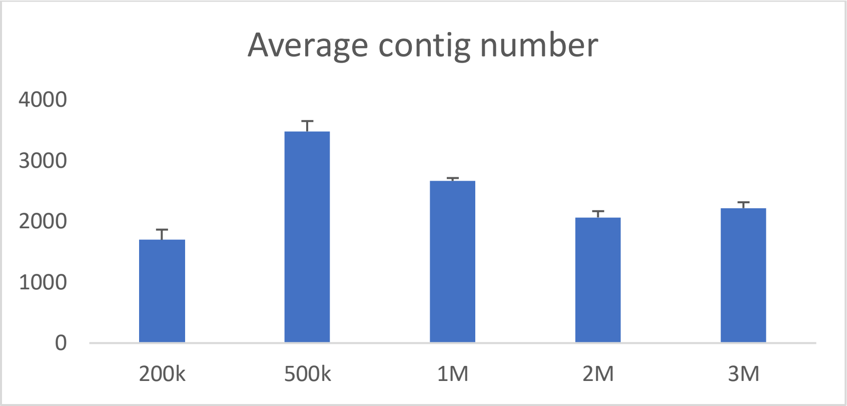

B.

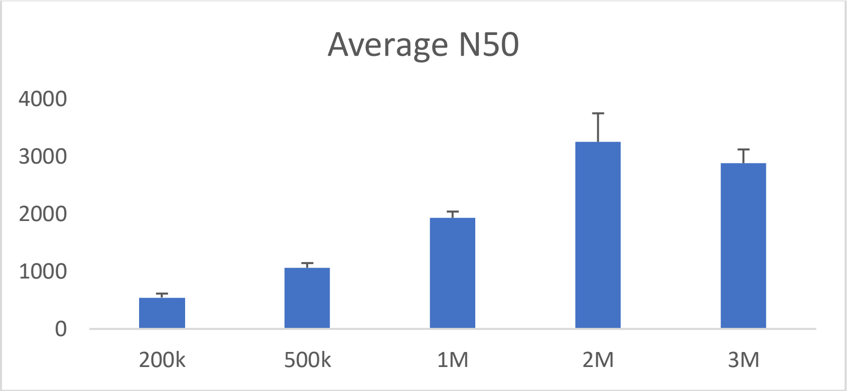

C.

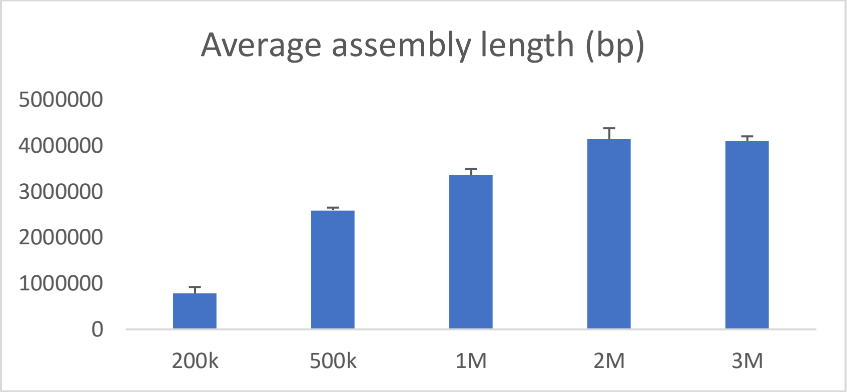

Supplemental Figure S2. Impact of starting read count on *De novo* assembly contig metrics. Metrics were generated for a test dataset (n=5) of randomly sampled, starting read counts: 200,000 (200k), 500,000 (500k), 1 million (1M), 2 million (2M) and 3 million (3M). A) Average number of contigs generated for each set of subsampled reads. B) Average N50 length (in bp) for each set of subsampled reads. C) Average length (bp) of contigs generated by *de novo* assembly.

Supplemental Table S5. Assembly statistics from Norman *et al.*, 2017 MHC reference haplotype Illumina short read dataset. *SRA:* SRP348947, *BioProject:* PRJNA764575.

Assembly metrics	OK649231	OK649232	OK649234	OK649235	OK649236
Number of reads	266995	217603	304342	210039	252657
Contigs (n)	1506	2227	1147	2425	1585
N50	4055	2549	6191	2274	3964
Max contig (bp)	24727	18382	36889	16305	23271
Total assembly length (bp)	4127329	3908195	4539318	3710459	4196340

Assembly metrics	OK649231	OK649232	OK649234	OK649235	OK649236
Number of reads	266995	217603	304342	210039	252657
Contigs (n)	1506	2227	1147	2425	1585
N50	4055	2549	6191	2274	3964
Max contig (bp)	24727	18382	36889	16305	23271
Total assembly length (bp)	4127329	3908195	4539318	3710459	4196340

Supplemental table S6. Reference MHC coverage with contigs generated from reads that mapped to reference genome

MHC Haplotype	% of haplotype covered	Total haplotype covered (bp)	Average % Identity	Median % Identity
APD	72	3591688	99.6	100
DBB	59.48	3002824	99.7	100
MANN	80	4022500	99.53	100
QBL	60.51	2968060	99.37	100

Supplemental Table S7. Reference MHC coverage with contigs generated from reads that did not map to initial reference genome.

MHC Haplotype	% Haplotype covered	Total haplotype coverage (bp)	Average % Identity	Median % Identity
APD	0.078	3872	99.8	100
DBB	0.140	7153	99.8	100
MANN	0.160	8233	99.83	100
QBL	0.053	2611	99.54	100

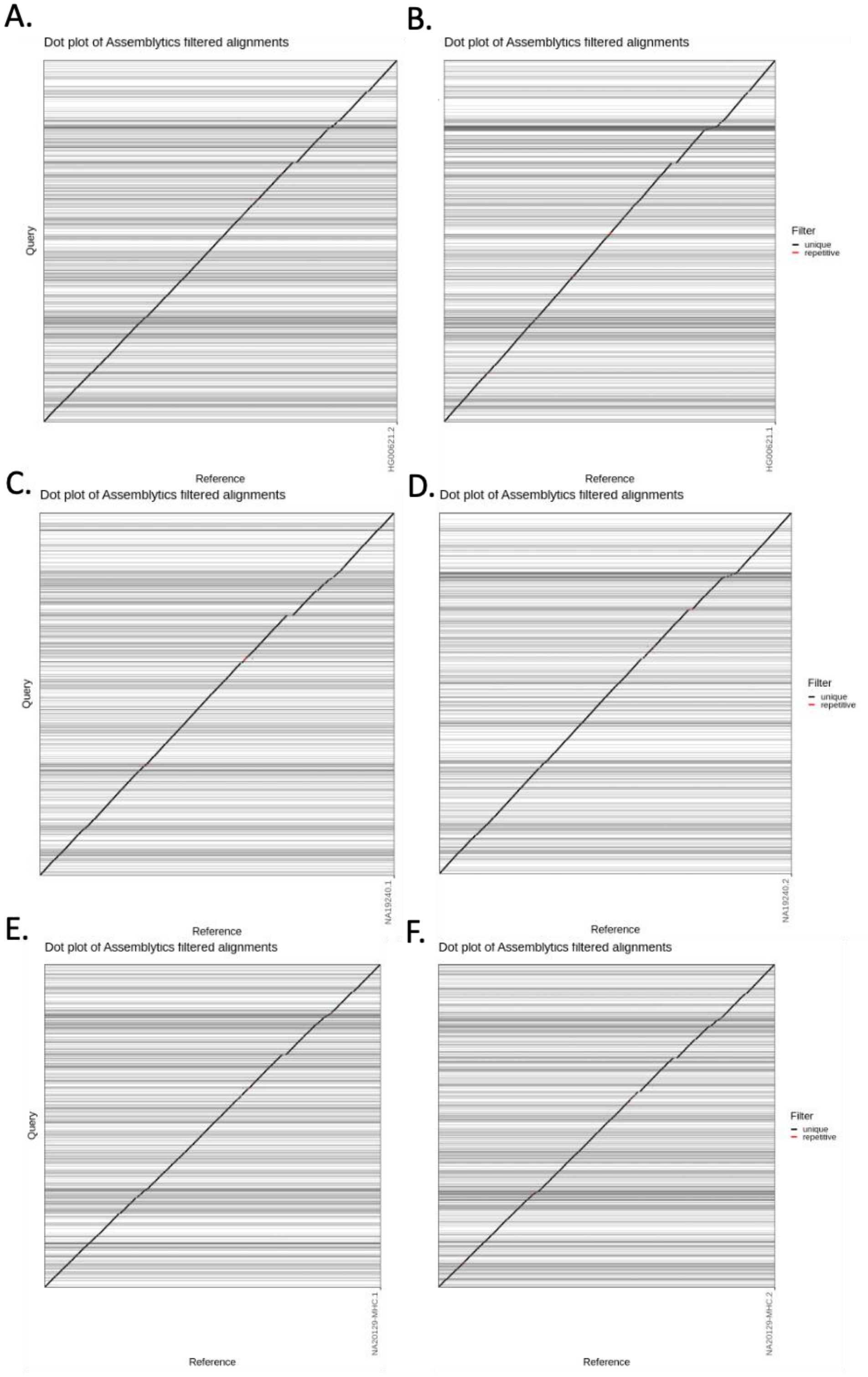

Supplemental Figure S3. Dotplot of *Assemblytics* assembly alignments between 1KGP WGS MHConstructor assemblies (y-axes) and their corresponding, phased reference MHC haplotypes (x-axes). The assemblies shown represent: HG00621 MHC haplotype A (A), HG00621 MHC haplotype B (B), NA19240 MHC haplotype A (C), NA19240 MHC haplotype B (D), NA20129 MHC haplotype A (E), NA20129 MHC haplotype B (F).

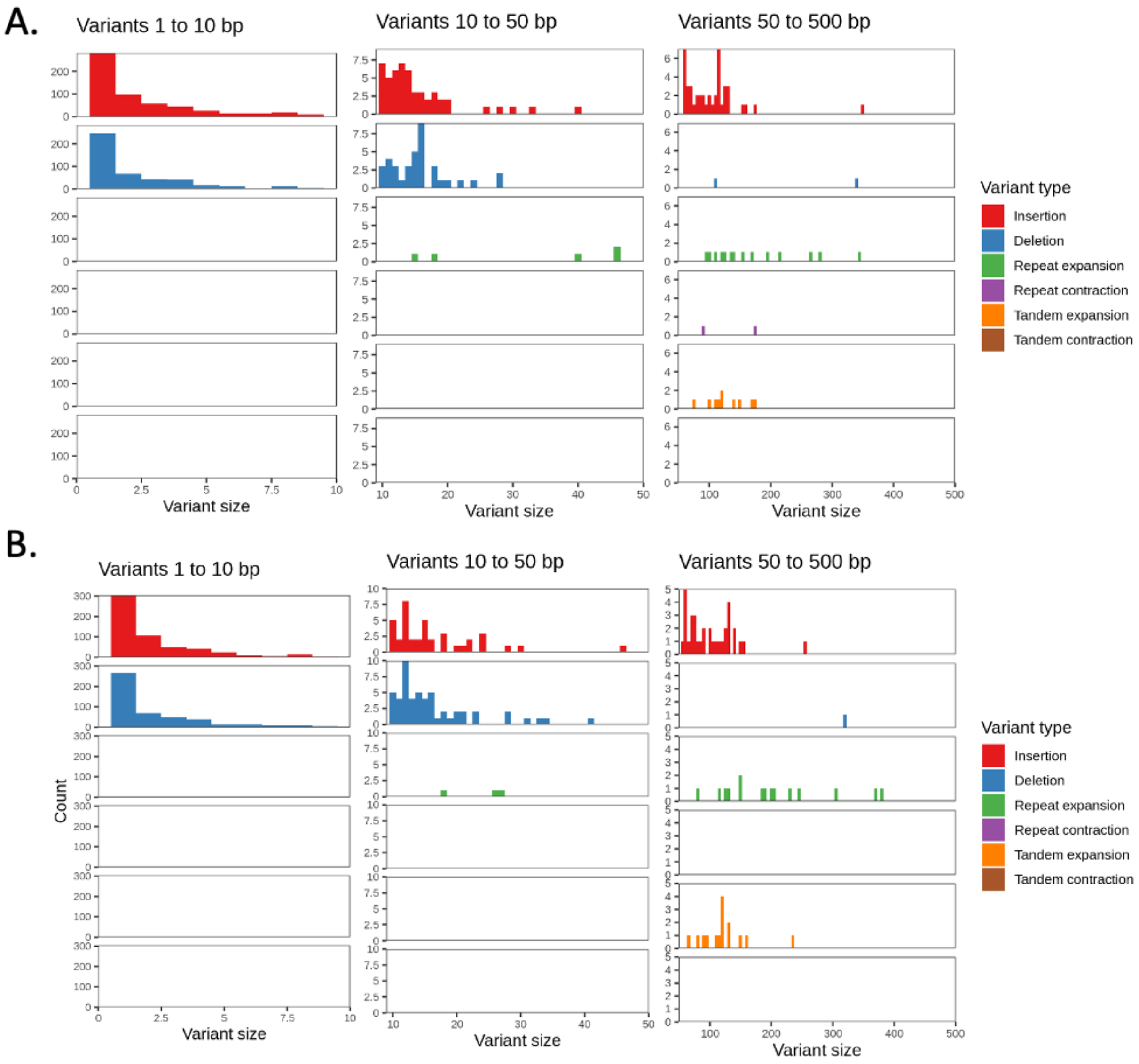

Supplemental Figure S4. *Assemblytics* analysis of false SV composition for the MHConstructor *de novo* assembly of HG00621, as compared to phased, reference MHC haplotypes for HG00621. A) HG00621 MHC haplotype A, B) HG00621 MHC haplotype B. Categories of assembly SV errors described in legend.

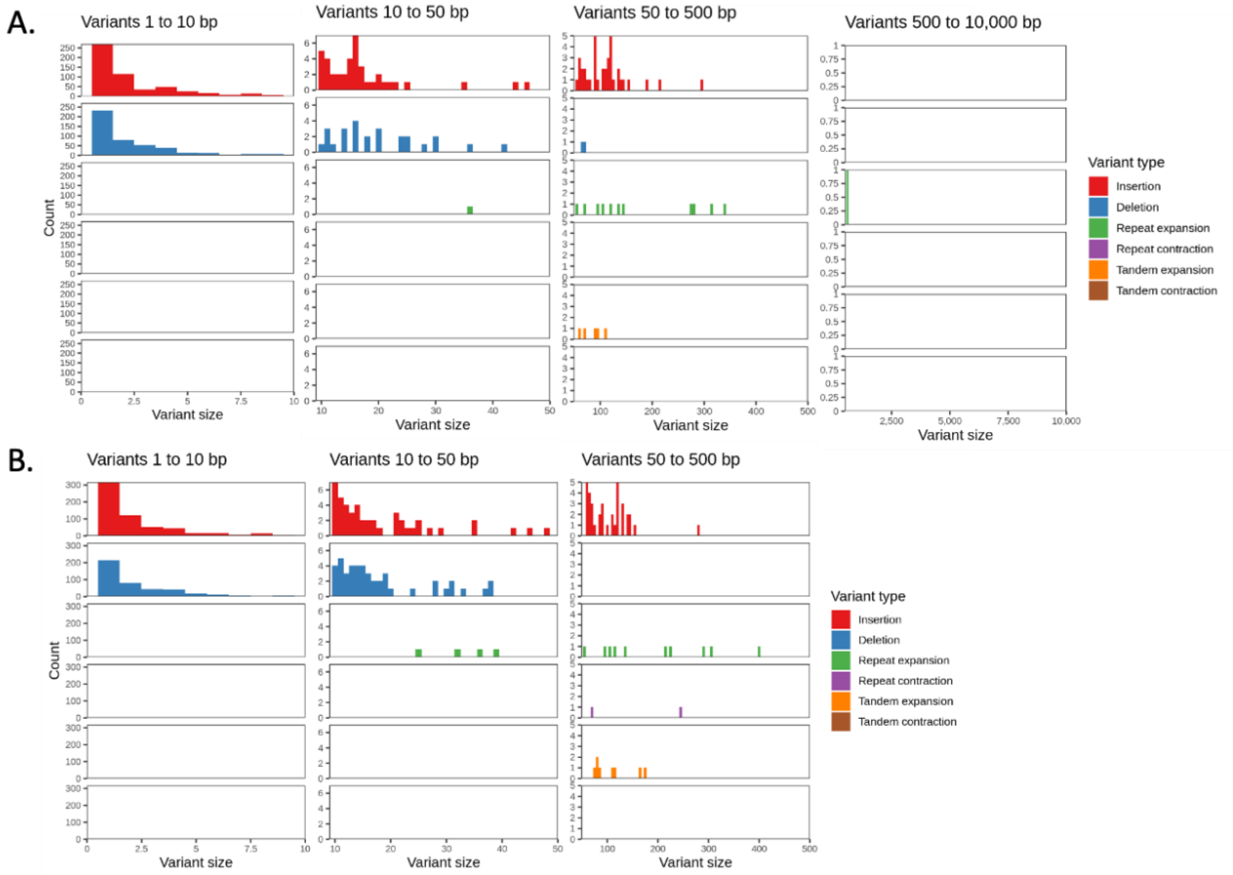

Supplemental Figure S5. *Assemblytics* analysis of false SV composition for the MHConstructor *de novo* assembly of NA19240, as compared to phased, reference MHC haplotypes for NA19240. A) NA19240 MHC haplotype A, B) NA19240 MHC haplotype B. Categories of assembly SV errors described in legend.

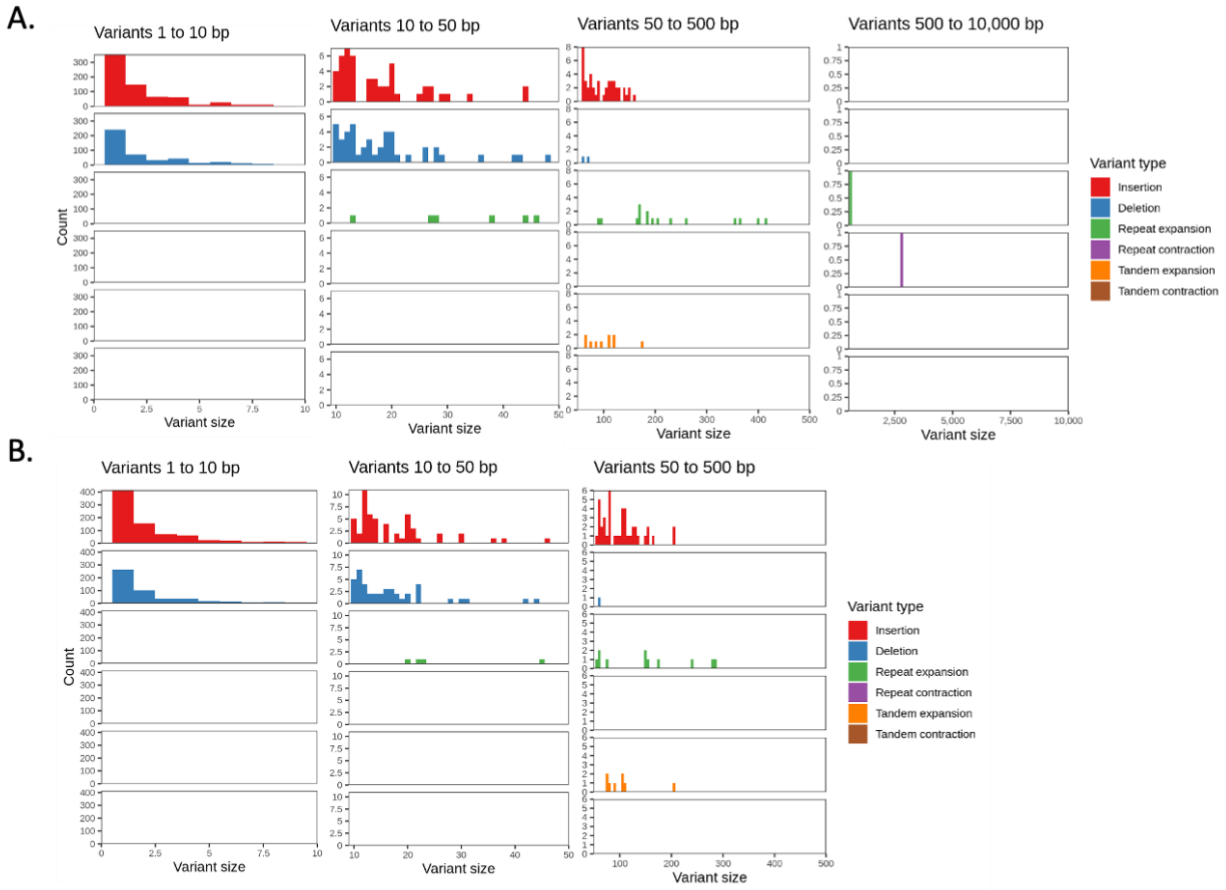

Supplemental Figure S6. *Assemblytics* analysis of false SV composition for the MHConstructor *de novo* assembly of HG00621, as compared to phased, reference MHC haplotypes for NA20129. A) NA20129 MHC haplotype A, B) NA20129 MHC haplotype B. Categories of assembly SV errors described in legend.

Supplemental Table S8. Number of error sites in base5-validated *de novo* assemblies that are caused by inclusion of a heterozygous site belonging to the alternative haplotype and those caused by assembly error.

Site category	HG00621.1	NA19240.1	NA20129.1
Heterozygous sites	5167	4602	5728
Assembly error sites	3068	5447	3021

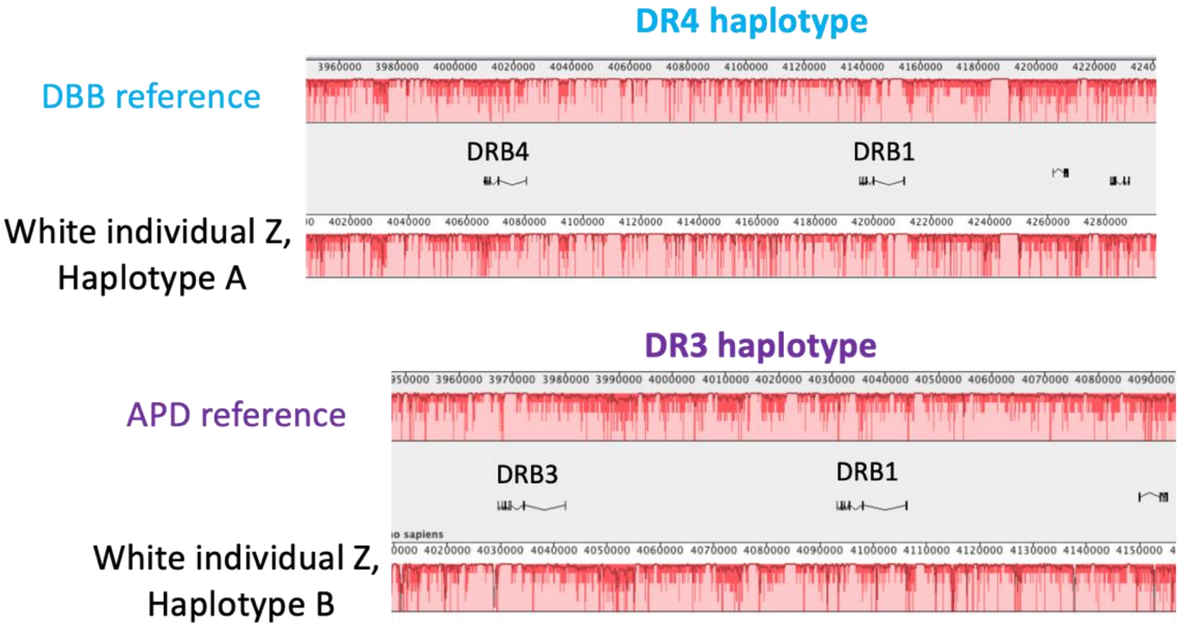

Supplemental figure S7. Mauve alignment plots confirm that the two DR haplotypes for heterozygous Individual Z are correctly assembled. In the top plot, the Individual Z sequence corresponding to the DR4 haplotype is displayed aligned against the DBB (DR4) published reference sequence (Houwaart *et al.*, 2022). In the bottom plot, the Individual Z sequence corresponding to the DR3 haplotype is displayed aligned against the APD (DR3) published reference sequence (Houwaart *et al.*, 2022). Light red indicates perfect alignment, dark red indicates presence of substitutions. HLA-DRB genes are labelled above their annotated coding regions.

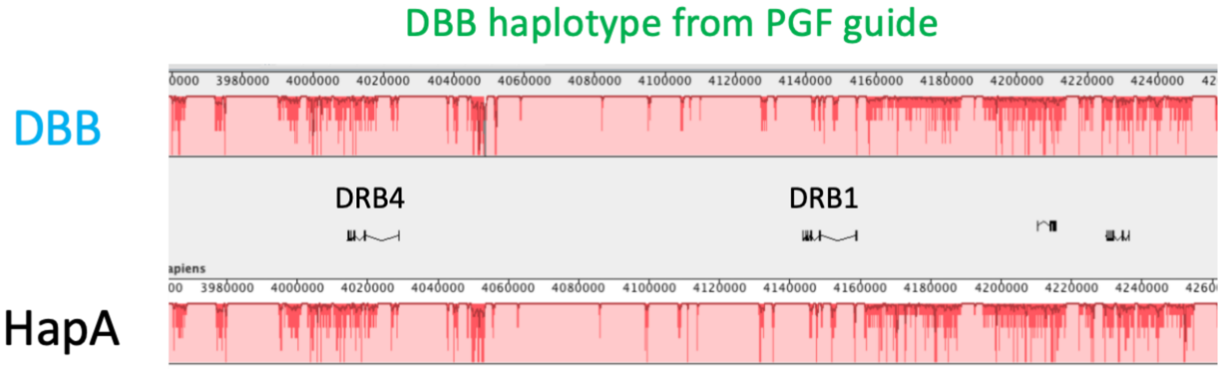

Supplemental Figure S8. HLA Class II structure can be correctly assembled even when an incorrectly-match haplotype is used as the assembly guide, so long as the correct haplotype is used for scaffolding orientation.

Supplemental Table S9. RepeatMasker annotation of repetitive sequence content in an MHConstructor WGS assembly and its corresponding phased MHC haplotype.

MHConductor NA19240.1

GC level: 44.77 %
bases masked: 2744988 bp (53.95 %)

	number of elements*	length occupied	percentage of sequence
SINEs:	3396	821766 bp	16.15 %
ALUs	2798	728111 bp	14.31 %
MIRs	592	92718 bp	1.82 %
LINEs:	1731	1050336 bp	20.64 %
LINE1	1134	848383 bp	16.67 %
LINE2	524	181556 bp	3.57 %
L3/CR1	51	12950 bp	0.25 %
LTR elements:	992	617160 bp	12.13 %
ERVL	228	171551 bp	3.37 %
ERVL-MaLRs	358	152149 bp	2.99 %
ERV_classI	295	215025 bp	4.23 %
ERV_classII	47	56590 bp	1.11 %
DNA elements:	700	174889 bp	3.44 %
hAT-Charlie	396	93293 bp	1.83 %
TcMar-Tigger	151	54356 bp	1.07 %
Unclassified:	21	13236 bp	0.26 %
Total interspersed repeats:		2677387 bp	52.62 %
Small RNA:	87	7042 bp	0.14 %
Satellites:	4	3266 bp	0.06 %
Simple repeats:	991	45653 bp	0.90 %
Low complexity:	191	11161 bp	0.22 %

Base5 NA19240.1

GC level: 44.90 %
bases masked: 2895112 bp (57.11 %)

	number of elements*	length occupied	percentage of sequence
SINEs:	3446	849271 bp	16.75 %
ALUs	2828	751675 bp	14.83 %
MIRs	611	96462 bp	1.90 %
LINEs:	1761	1122194 bp	22.14 %
LINE1	1138	906985 bp	17.89 %
LINE2	545	193494 bp	3.82 %
L3/CR1	57	14535 bp	0.29 %
LTR elements:	1019	649642 bp	12.81 %
ERVL	232	177122 bp	3.49 %
ERVL-MaLRs	370	160049 bp	3.16 %
ERV_classI	299	228338 bp	4.50 %
ERV_classII	48	61061 bp	1.20 %
DNA elements:	728	182701 bp	3.60 %
hAT-Charlie	413	97084 bp	1.91 %
TcMar-Tigger	149	56228 bp	1.11 %
Unclassified:	20	21501 bp	0.42 %
Total interspersed repeats:		2825309 bp	55.73 %
Small RNA:	90	7288 bp	0.14 %
Satellites:	7	3455 bp	0.07 %
Simple repeats:	1013	46435 bp	0.92 %
Low complexity:	202	12146 bp	0.24 %

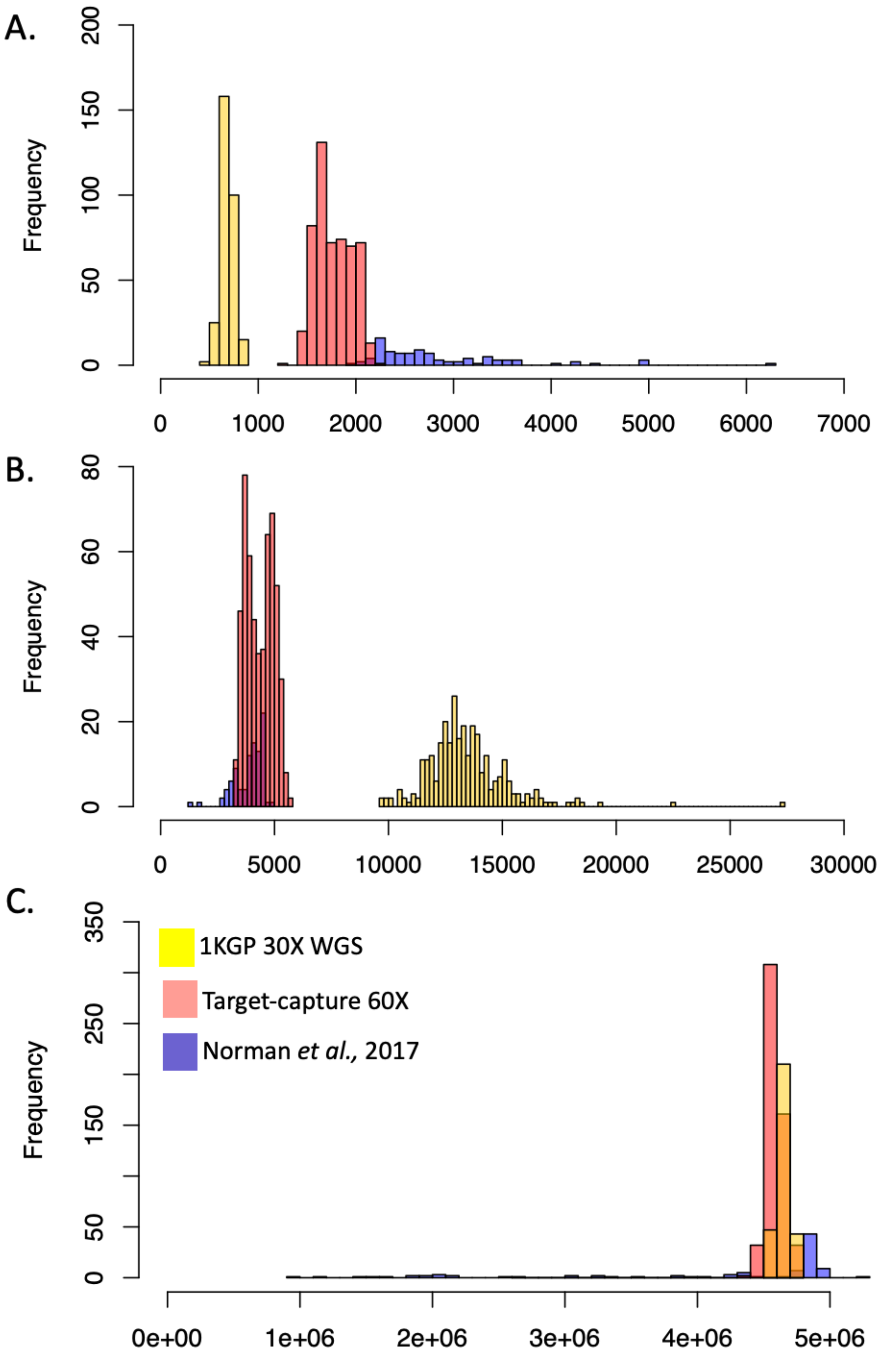

Supplemental Figure S9. MHConstructor assembly metrics for target capture and WGS assemblies. Purple distributions represent published results from Norman *et al.*, 2017, n=95 haplotypes. Pink distributions represent assemblies generated from target-capture, 60x short read data, n=536 haplotypes. Yellow distributions represent assemblies generated from 1000Genomes WGS 30x short read data, n=300 haplotypes. A) Histogram of the number of contigs per assembly. B) Histogram of assembly N50 (bp), per assembly. C) Histogram of assembly lengths (bp).

.

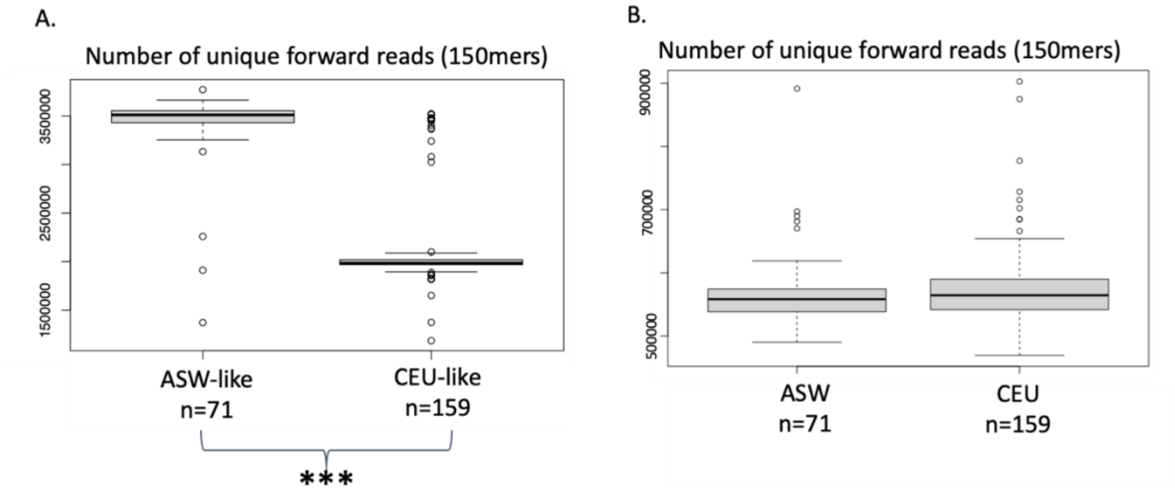

Supplemental Figure S10. Nucleotide diversity within sequencing reads. A) Number of unique forward reads in subsampled ASW-like and CEU-like individuals, from target-capture approach. B) Number of unique forward reads in ASW and CEU populations, from WGS approach.
